# Development of the Brazilian Version of a Pan-Canadian Behavior Change Program and Its Health and Fitness Outcomes

**DOI:** 10.3390/jcm11195926

**Published:** 2022-10-08

**Authors:** Juliano Schwartz, Paul Oh, Shannon S. D. Bredin, Ryan E. Rhodes, Maira B. Perotto, Alejandro Gaytán-González, Darren E. R. Warburton

**Affiliations:** 1 Physical Activity Promotion and Chronic Disease Prevention Unit, University of British Columbia, Vancouver, BC V6T 1Z4, Canada; 2 Cardiovascular Prevention and Rehabilitation Program, Toronto Rehabilitation Institute, University Health Network, Toronto, ON M4G 1R7, Canada; 3 School of Exercise Science, Physical and Health Education, University of Victoria, Victoria, BC V8P 5C2, Canada; 4 West Toronto Diabetes Education Program, LAMP Community Health Centre, Etobicoke, ON M8Z 1K2, Canada; 5 Institute of Applied Sciences for Physical Activity and Sport, University of Guadalajara, Guadalajara 44430, Mexico

**Keywords:** prevention, chronic disease, Canada, Brazil

## Abstract

Chronic diseases are a major health problem worldwide, especially in lower-income jurisdictions. Considering this scenario, the World Health Organization has recently established, as a research priority, preventive interventions for populations from lower-income countries, such as the middle-income country of Brazil. The purpose of this article is to describe the components of a pan-Canadian lifestyle program adapted to Brazilians and to report its health and fitness outcomes. A 12-week program called ACCELERATION was translated and culturally adapted to Brazilians. A quasi-randomized controlled trial was designed, consisting of weekly emails and educational videos addressing risk factors for chronic disease. Health and fitness measures included body composition, cardiovascular variables, aerobic fitness, and muscular strength. The Brazilian experimental group showed maintenance in heart rate, blood pressure, and VO_2_max values while presenting an improvement of 3.3% in body fat percentage (*p* = 0.040, *d* = −0.325) and 5.1% in muscular strength (*p* = 0.039, *d* = 0.328). Overall, these results were similar to the Canadian intervention. Based on these findings, the Brazilian version of the program has the potential to contribute to the fight against chronic diseases in Brazil.

## 1. Introduction

Preventable behavioral risk factors for chronic medical conditions, such as physical inactivity, low-quality diet, smoking, and excessive alcohol intake, have received increased attention from governments and other organizations across the globe [1,2,3]. Although these risk factors affect broad populations in both high- and lower-income countries, those living in low-income settings are more severely impacted by these chronic medical conditions due to significant socioeconomic disparities [4]. As pointed examples, most residents in these areas cannot afford the time and money to be physically active on a regular basis, and/or frequently face daily challenges to follow a healthy diet [5]. Further, public services provided by healthcare professionals are often scarce in lower-income regions, and the cost of private healthcare is often prohibitive [6]. Thus, there is a need for innovative preventative health interventions to be delivered through alternative channels, in order to reach individuals in these situations [7]. Additionally, most research aimed at generating strategies to tackle the problems related to chronic medical conditions and their risk factors has been conducted with populations from high-income countries [8]. Given the considerable socioeconomic differences between high- and low- and middle-income countries (LMICs), however, intervention designs and outcomes from studies with high-income countries’ populations are not appropriate to implement directly on populations from lower-income countries [9,10,11]. Accordingly, the World Health Organization has recently recommended, as a research priority, behavior change interventions with populations from LMICs to compare the results with those conducted on high-income countries’ populations [12]. The Organization highlights that such initiatives are necessary to reduce health disparities and increase the effectiveness of public health recommendations in lower-income regions [13]. 

With the substantial recent advances in telecommunications and video technology, access to information has increased exponentially [14]. These advances have led to the development of more affordable electronic devices—such as computers, tablets, and smartphones—which have allowed the majority of households to have at least one device connected to the internet, even in economically disadvantaged communities, contributing to the swift sharing of information [15]. However, while most people are aware of the numerous benefits of healthy and active lifestyles [16,17], many individuals do not engage in positive health behaviors [18,19]. Two possible reasons for this lack of engagement are that initiatives to tackle unhealthy behaviors might not be sufficiently appealing to some individuals, for example, for not considering personal preferences, and the fact that some individuals simply do not have access to opportunities aimed at health behavior change, mainly in LMICs [20,21,22,23].

To bridge these gaps, a 12-week pan-Canadian intervention aimed at chronic disease prevention through health behavior change was adapted to be delivered to Brazilians. Besides cultural adaptions, other modifications (particularly considering the appropriate socioeconomic context) were made and are presented in the following sections. These modifications are related to the mode of delivery and the incorporation of some behavior change strategies that were presented in studies published after the delivery of the original program. Such strategies addressed mainly affective judgements and perception of opportunities to adopt healthier lifestyles, which have been shown to be essential to translate intentions into behaviors [24,25,26]. Otherwise, the adapted version was very similar to the Canadian protocol. The planning and delivery of the original intervention took place between 2013 and 2016, and the project was named the ACCELERATION (activity, smoking cessation, healthy eating and alcohol education, intervention, and motivation) program, which targeted individuals at risk of developing chronic diseases [27]. The Canadian project was implemented in four provinces at the following locations: Cardiovascular Prevention and Rehabilitation Program, Toronto Rehabilitation Institute—University Health Network in Ontario; Montreal Behavioral Medicine Centre—L’Hôpital du Sacré-Cœur de Montréal in Quebec; Physical Activity Promotion and Chronic Disease Prevention Unit as well as the Indigenous Health and Physical Activity Program—University of British Columbia in British Columbia; and Community Cardiovascular Hearts in Motion in Nova Scotia [28]. These centers have many years of experience in primary and secondary prevention of chronic medical conditions and were well structured to provide in-person education and exercise sessions in every week of the original project.

Another manuscript about the Canadian program, addressing changes in behavioral risk factors for chronic disease, is under submission. In the present publication, we describe the aspects involved in the development of the Brazilian version of the ACCELERATION program and report its health and fitness outcomes. Here, we present the components of the adapted program and how the outcomes were assessed, in order to make this intervention also available to the Brazilian population. The novelty of this program was such that it coupled newer behavior change frameworks and technologies to enhance the health and wellbeing of individuals from LMICs at risk of developing chronic disease, directly addressing the World Health Organization’s call for behavioral intervention research with this population [12,13].

The primary hypothesis of the study was that the Brazilian intervention would lead to significant improvements in health and fitness outcomes. A secondary hypothesis was that the Brazilian experimental group would present similar results to the Canadian group and better results than the control group.

## 2. Materials and Methods

The intervention was based on different behavior change strategies used in chronic disease prevention and physical activity promotion centers in the Canadian provinces where the original program was delivered [24,29,30,31,32]. Keeping the main aspects of the Canadian protocol, the Brazilian version incorporated cultural adaptations and modifications considering the appropriate socioeconomic context. 

All materials and components of the ACCELERATION program were translated and culturally adapted into Brazilian Portuguese by a certified translator and a Brazilian-Portuguese-speaking physiotherapist, who has advanced clinical exercise physiology training and is familiar with chronic disease prevention programs delivered in Brazil and Canada. The Brazilian version of the program was delivered by this physiotherapist in Vancouver, Canada, with a team of Brazilian health professionals (physician, dietitian, psychologist, physiotherapist, and kinesiologist) who were also practitioners in Canada and collaborated with the project by providing their input to the Brazilian version of the intervention and addressing specific questions of the participants during the program. 

Although the structure of the Brazilian project was based on the original Canadian program, participants’ feedback to the latter suggested that some adjustments would improve the practicality of the translated and adapted program. The senior researchers who contributed to the development of the Brazilian program came to an agreement to reduce the number of questionnaires to be applied in the initial and follow-up assessments, to encourage the full completion of these documents. 

There was also an agreement to remove excessive alcohol intake as an inclusion criterion, given the low number of participants presenting such behavior in the original intervention. Thus, the Brazilian version of the program was known as ACCELERATION (activity, smoking cessation, healthy eating education, intervention, and motivation).

### 2.1. Study Design 

Participants in the BE and BC cohorts were recruited from the province of British Columbia and living in and around the Greater Vancouver area. According to the Canadian population census [33], in 2016, Brazilians made up only 0.1% of the 2,264,823 people living in the Greater Vancouver area. Additionally, many of these individuals did not live in the city of Vancouver, but rather in other municipalities of the greater metropolitan area. Therefore, to maximize the project’s reach, the program was tailored to allow participation during the 12 weeks without the need to travel. Apart from the assessments before and after the intervention, participants were able to follow the program wherever and whenever was suitable for them. Considering also that the daily routines of potential participants varied widely, the intervention was delivered online.

An open-label quasi-randomized controlled trial was used, with the initial Brazilian participants being assigned to the Brazilian experimental group (BE) and the other participants being assigned to a waitlist Brazilian control group. At baseline, both groups completed health and fitness assessments. Following these initial evaluations, the intervention was delivered to the experimental group, and participants in the control group were asked to maintain their current lifestyles without any change during that period. After the 12-week intervention, both groups completed the health and fitness assessments again. The control group then received the intervention. The results of the BE cohort compared to the results of the BC cohort the Canadian experimental group (CE). There was no control group in the Canadian intervention, which was designed as a pre- vs. post-intervention within-subject evaluation rather than a randomized controlled trial. Participants in the CE cohort all had risk factors for chronic disease (i.e., physical inactivity, poor diet, or smoking) and were recruited in the four provinces as described in the Introduction section. Persons in British Columbia and Nova Scotia came mainly from workplace settings, while those in Quebec were members of a community center, and most of the Canadian participants were from Ontario, where they were identified from healthcare-affiliated settings.

The assessments of the adapted program for the BE and BC groups were carried out at the Physical Activity Promotion and Chronic Disease Prevention Unit at the University of British Columbia. The study followed the Declaration of Helsinki, and the project was approved by the Clinical Research Ethics Board of the University of British Columbia (H17-03564). All participants provided written informed consent prior to research participation.

### 2.2. Inclusion Criteria

To be included in the study, participants had to be Brazilians (living in the Greater Vancouver area), over 18 years old, who spoke Brazilian Portuguese fluently and presented at least one of the following criteria: (1) physical inactivity (<150 min of moderate-to-vigorous physical activity (MVPA) per week), (2) unhealthy diet (consumption of fewer than five fruits/vegetables per day), and/or (3) smoking (any amount of personal tobacco use by self-report). Participants could present with a medical co-morbidity, provided the condition was stable. All participants needed to have consistent internet access to be able to watch educational videos and to communicate over email with the research team. Such access could be through the use of computers, tablets, or smartphones.

### 2.3. Exclusion Criteria

Participants were excluded from the study if: (1) they presented with an unstable clinical condition, (2) were unable to participate fully in the program due to mental or physical limitations, and/or (3) were concurrently participating in any other study including intensive health behavior modification.

### 2.4. Participant Recruitment 

In partnership with the Brazilian consulate in Vancouver, men and women were invited to participate in the program through different mechanisms. These included emails and social media postings via official channels. The program was also promoted in activities for Brazilians living in Vancouver and neighboring cities, such as lectures about an active lifestyle, as well as distributing information leaflets at community events. 

### 2.5. Behavior Change Intervention 

#### 2.5.1. Motivational Interviewing

The first phase of the intervention consisted of an interview, which was structured following a motivational communication framework, and focused on getting a broader perspective of each participant’s motivation and confidence to change health behavior [34]. This approach was used to strengthen motivation and confidence for health behavior change, and to increase the participants’ commitment to their goals, by eliciting and exploring their reasons for change, through acceptance and compassion [35].

This participant-centered collaborative communication provided program support in a goal-oriented style. Without approving or disapproving of the participants’ behavior, a partnership was established with each individual to evoke the skills and strengths they already had [34]. Together with the researcher, each participant made decisions on goals and how to achieve them. Open-ended questions to encourage elaboration, with affirmations to acknowledge the participant’s experiences and reflective listening to demonstrate empathy and interest, were followed by summary statements to confirm the understanding of the information shared by the participant [36]. Focusing on the positive aspects involved in engaging in healthier lifestyles, rather than underscoring the perils of poor health behaviors, each individual was encouraged to consider their perceptions about the advantages and disadvantages involved in adopting positive health behaviors [37].

The final part of the motivational interviewing was dedicated to action planning and goal setting [38], based on the results of the baseline assessments. Considering what was relevant for them, each participant received assistance to develop a specific, attainable, and detailed plan for short- and long-term moderately challenging goals, to be measured throughout the intervention and at the end of the program. Participants were oriented to include the following in their plans for each goal: what, how, where, when/how often, and how much [39]. These individual goals and plans/strategies were revised in different phases of the program. Although participants were encouraged to set both short- and long-term goals, the emphasis was on the former, to promote a perception of control over the situation and to build self-efficacy towards achieving long-term goals [40]. These approaches consisted of strategies from the taxonomy of behavior change techniques developed by Michie et al. in 2013, namely goal setting (behavior) and action planning [41].

#### 2.5.2. Structured Online Educational Program

The online sessions consisted of a structured education intervention as well as different behavior change techniques emphasizing self-management and motivation [42,43,44]. Participants received individualized physical activity and dietary recommendations, based on the pre-intervention assessments. Each participant also received an elastic resistance band appropriate to their strength level and was oriented on how to recognize whether another band would be necessary (based on an increase in musculoskeletal fitness and/or damage to the equipment), and on how to proceed. Additionally, participants were instructed to monitor their daily steps during the 12 weeks using a free mobile phone app (Pacer). 

Based on the participants’ pre-intervention health and fitness assessments, the exercise prescription included a 12-week program consisting of progressive aerobic and strength activities. To encourage autonomy, participants were advised to follow the exercise program where convenient, including parks, gyms, and their own homes. 

The education sessions consisted of videos focusing on health behaviors, delivered via weekly emails. The theme and content addressed in each video as well as the behavior change techniques and respective mechanisms of action are shown in Table 1. Additionally, more personalized guidance was provided during individual interactions throughout the program, using specific techniques/mechanisms of action [24,45,46], addressing the topics presented in Table 2.

The videos had an average duration of seven minutes. At the end of each video, there was a question that the participants were asked to answer by email. This served to check whether the participants watched the video and their understanding of the topic. When necessary, additional support and/or clarification was provided. 

Throughout the intervention, participants also received a short, individualized email every week to increase self-efficacy and motivation. These emails addressed long-term benefits of better lifestyles, as well as immediate/short-term ones, focusing on positive emotions and overall wellbeing [47,48]. Moreover, the emails provided additional resources to facilitate the adoption of healthy behaviors and also served to address any concerns that could have arisen throughout the program. The theme addressed each week varied according to each participant’s specific needs, and therefore, several content areas were addressed more than once. However, overall, they followed the main topics presented in Table 2. 

### 2.6. Assessment Measures 

All measures were collected before and after the intervention, except for demographic characteristics, which were collected only at the beginning of the program.

#### 2.6.1. Pre-Participation Physical Activity Screening

All participants first completed the Brazilian version of the PAR-Q+, for physical activity clearance [49]. 

#### 2.6.2. Demographics

At baseline, the following demographic characteristics were collected: age, sex, diagnosed chronic diseases, income, employment, marital status, time to travel to the assessments, and mode of transportation to travel to the assessments.

#### 2.6.3. Health and Fitness Measures

Assessments of health and fitness included: body composition, cardiovascular measures, and physical performance. For body composition, participants were instructed to wear light clothes and remove shoes, socks, and any other belongings, such as keys and wallets. Height was measured with a stadiometer (Seca 213, Hamburg, Germany) to the nearest 0.1 cm; weight and body fat were recorded using a bioimpedance scale (Tanita TBF-300, Arlington Heights, IL, USA), to the nearest 0.1 kg and 0.1%, respectively; and waist circumference (WC) was measured immediately above the lateral border of the iliac crest, with a standard anthropometric tape (Seca 200, Hamburg, Germany), to the nearest 0.1 cm. The following formula was used to determine the body mass index (BMI): weight in kg divided by height in meters squared [50].

After 5 min of rest in the seated position, heart rate (bpm) as well as systolic blood pressure and diastolic blood pressure (mmHg) were recorded three times at one-minute intervals, using an automated measurement system (BP-Tru, Coquitlam, BC, Canada). The average of the two last measures for each variable was adopted [51,52]. 

Regarding physical performance, handgrip strength was measured in kg, twice in each hand, with a one-minute interval between the assessments, using an analog dynamometer (Almedic, Montreal, QC, Canada). The sum of the highest measure of each hand was adopted [53]. Further, a submaximal six-minute walk test (6MWT) was used to determine aerobic fitness. Participants were asked to walk back and forth, as fast as possible, in a 20 m corridor. The walked distance was recorded, and the maximal oxygen consumption (VO_2_max) in mL·kg^−1^·min^−1^ was estimated with the following formula: 70.161 + (0.023 × walked distance (m)) − (0.276 × weight (kg()) − (6.79 × sex, where male = 0, female = 1) − (0.193 × resting heart rate (bpm)) − 2 (0.191 × age (years)) [54].

### 2.7. Adaptation Process

The steps involved in adapting the program to Brazilians included the use of plain language, to be understood by individuals of different ages and regions; the replacement of images, to show contexts that look familiar to Brazilians; the replacement of cultural references to ensure relevance to these individuals; and the use of relevant, evidence-based content, to make the program appealing for the audience. The adaptation process included the recommendation of physical activities that were considered more attractive/enjoyable by this population, such as martial arts for quality of life and health purposes, rather than competition [55,56], and the development of the Brazilian version of the Physical Activity Readiness Questionnaire for Everyone (PAR-Q+). The Brazilian PAR-Q+ is a valid and evidence-informed tool, specifically tailored for this population, to facilitate a safe engagement in physical activity [49]. The instrument has an excellent internal consistency (0.993) and a good to excellent reliability (0.901, 95% CI: 0.887–0.914) [49]. In terms of diet, this component of the program was based mainly on the latest Brazilian dietary guidelines [57], which were a pioneer evidence-based food guide with a revolutionary and wholistic approach to informing healthy eating. These guidelines emphasize the importance of unprocessed over ultra-processed foods and address the context of eating rather than the usual focus on nutrients and food groups [20,58,59]. More details on the cultural adaptation of the intervention are presented in Table 3.

### 2.8. Statistical Analysis 

The outcome measure for statistical planning purposes of the original ACCELERATION program was change in physical activity. The Canadian protocol aimed to double the proportion of individuals engaging in ≥150 min of MVPA per week. At the time of the planning of that intervention, Statistics Canada estimated that 15% of the adult population was meeting the international guidelines of ≥150 min of MVPA per week [60]. The original protocol projected to double this proportion to 30%. Using a 1-sided *t*-test to compare binomial proportions with an alpha of 0.05 and power of 0.80, the required sample size would be a minimum of 95 individuals. According to the Brazilian database Vigitel, an equivalent of Statistics Canada, 35% of the Brazilian population were meeting these guidelines when the Brazilian version of the program was being planned [61]. Using the same method, the required sample size for each Brazilian group was estimated at 23 participants.

Preliminarily, data were examined for accuracy and detection of missing values. Data were analyzed only when collected at baseline. For data missing due to loss to follow-up, Little’s test was used to determine whether data were missing completely at random throughout the dataset rather than revealing a systematic pattern. The Little’s test confirmed that data were missing completely at random, which supported expectation maximization imputation. Variables with missing data had no more than 5% of missing values, which were handled using the expectation-maximization algorithm [62,63].

Subsequently, data were analyzed for participants with complete pre- and post-assessments and carried out for each variable. Ratio variables (except age) were analyzed using a two-way repeated measures analysis of covariance (ANCOVA) with group by time design (3 × 2) adjusted for age, sex, marital status, income, and employment. The post hoc analyses included adjustments following the Bonferroni method. Age was compared among groups by using one-way ANOVA with Welch’s correction and Games–Howell as post hoc given that the variances were heterogeneous (Levene’s test *p* < 0.05). The comparison of categorical data among groups was performed using the chi-squared test of independence (X^2^) with multiple Z-tests for proportions with Bonferroni adjustments as post hoc analysis.

Effect sizes for group comparisons of ratio variables were calculated with omega squared (ω^2^) for ANCOVA, whereas pre–post comparison effect sizes were calculated with Cohen’s *d*. In categorical variables, phi statistic (φ) and Cramer’s V were calculated as effect size statistics. The reference cut points to categorize the effect sizes as small, medium, or large were: 0.01, 0.06, 0.14 for ω^2^; 0.2, 0.5, 0.8 for Cohen’s *d*; and 0.1, 0.3, 0.5 for φ and Cramer’s V [64,65]. Any effect size below the cut point for a small effect was considered trivial.

Continuous data were expressed as mean ± standard deviation or least squares mean ± standard error of the mean (SEM), whereas pre–post differences were reported as least squares mean (95% CI). Categorical variables were expressed as frequency counts (percentage). 

A result was deemed significant for a *p*-value < 0.05. All analyses were carried out in SPSS v.27, and graphs were drawn in GraphPad Prism v.7.04 for Windows.

## 3. Results

All Brazilian participants were recruited at the same time, and a total of 125 adults met the inclusion criteria. Out of those, 84 individuals attended the laboratory assessments. The first 46 participants were allocated to the BE group, and 38 who enrolled after the establishment of the experimental group were enrolled in the BC group. 

A total of eight participants withdrew from the study (*n* = five in BE; *n* = three in BC). Reasons for withdrawal included: changes in work/school workload and/or schedule and pregnancy. No participants withdrew from the program due to program-related adverse effects (according to self-report). A total of 41 participants in the BE group and 35 participants in the BC group completed the study. 

All individuals but one in each group were cleared to become more physically active, according to the PAR-Q+. These two individuals were directed to obtain clearance from their family physicians. The participant in the control group presented the physician clearance form, highlighting specific precautions, which were followed during the delivery of the program to this individual. However, the participant from the experimental group did not present such a form. Therefore, this individual agreed to proceed with dietary changes only, and although this participant received special attention during the whole program, no intervention nor any physical activity material was provided to them.

Although the original intervention was mainly focused on primary prevention populations across Canada, some participants from the Canadian program were recruited in healthcare centers. As a consequence, this sample had several older adults and individuals with diagnosed chronic diseases, whereas the Brazilian groups had no older adults and no participants with diagnosed chronic diseases. Moreover, since the Canadian intervention had excessive alcohol intake as an additional possible inclusion criterion, which was not the case for the Brazilian groups, participants from the original project with the characteristics above were not included in the analyses of the present study, aiming at more homogeneous groups for comparisons. 

**Figure 1:**
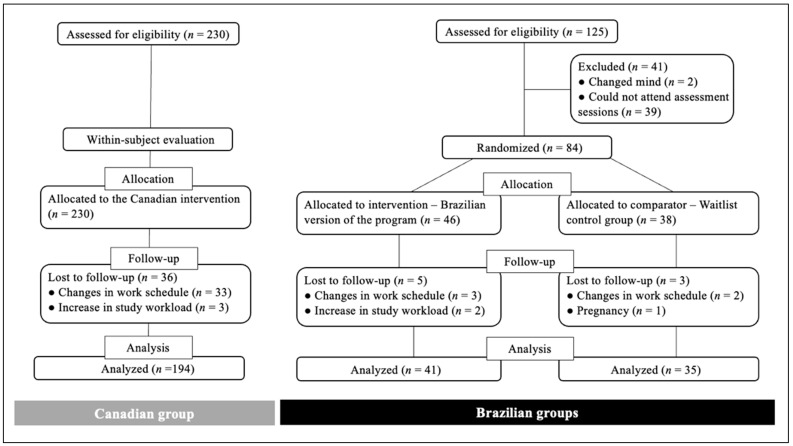
Flow of participants through the study.

A total of 230 Canadian adults, who were younger than 65 years, with no diagnosed chronic diseases, and reporting not consuming alcohol beyond the limits established by Canada’s low-risk alcohol drinking guidelines [66], attended the baseline assessments of the original project. Of those, 194 participants completed the original program. The flow of participants in each group through the study is shown in Figure 1.

For personal reasons, some participants in each group did not perform all assessments at baseline and/or after the 12 weeks. Therefore, the sample sizes of each group differ among variables.

### 3.1. Demographic Characteristics 

The socioeconomic characteristics of each group for sex, marital and employment status, income, as well as details about the access to the assessment’s facility, namely mode of transport and commute time, are presented in Table 4.

For all variables, significant differences were found between the Canadian and the Brazilian groups. The CE group was older than both Brazilian groups and had the highest female proportion, which was significantly higher than BE but not BC. The CE group had the lowest proportion of married participants, with a significant difference from BE but not BC. For income, the proportion of participants in CE earning >CAD 100,000 per year was significantly higher than BC but not BE. Conversely, both BE and BC had a higher proportion of participants earning <CAD 50,000 per year than CE. Regarding employment, more participants in CE were employed full-time, and fewer participants were unemployed in comparison to BE and BC. In terms of transport time to the facility in charge of the intervention, there were more participants in CE that spent <30 min to travel than the Brazilian groups, and a lower proportion of participants in CE spending >60 min in transport than BE and BC. For transport mode, there was a higher proportion of participants in CE using private transport than BC, and a lower proportion of participants in CE using public transit than both Brazilian groups.

### 3.2. Health and Fitness Measures

#### 3.2.1. Body Composition

**Figure 2:**
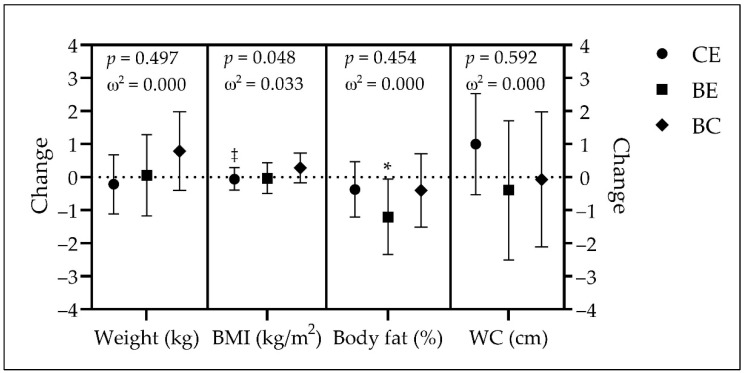
Changes over time in weight, BMI, body fat, and WC. Data expressed as least squares mean and 95% confidence intervals; *p*-values calculated with one-way ANCOVA for group comparisons (adjusted for age, sex, marital status, income, and employment); omega squared (ω^2^) as effect size. ^‡^ Denotes a significant difference versus BC (*p* < 0.05); * denotes a significant change over time (*p* < 0.05). BC: Brazilian control group (diamonds); BE: Brazilian experimental group (squares); BMI: body mass index; CE: Canadian experimental group (circles); WC: waist circumference.

The height of each group (mean ± SD) was as follows: 164.8 ± 8.2 cm in CE, 167.1 ± 9.9 cm in BE, and 169.0 ± 11.0 in BC. There were no significant differences among groups (*p* = 0.057; ω^2^ = 0.022). The other body composition measures of each group, at baseline and at the end of the intervention, are presented in Table 5. The differences over time for each group are presented in Figure 2.

There were no significant differences among groups for any body composition variable at any time point.

Participants in CE had no significant changes in weight (−0.2 kg, *p* = 0.638, *d* = −0.034), BMI (−0.1 kg/m^2^, *p* = 0.751, *d* = −0.023), body fat (−0.4%, *p* = 0.384, *d* = −0.064), and WC (1.0 cm, *p* = 0.199, *d* = 0.093) over time. A single significant change over time was observed in BE for body fat, with a decrease of 1.2% (*p* = 0.040, *d* = −0.325), whereas there was no significant change in weight (0.1 kg, *p* = 0.952, *d* = 0.015), BMI (−0.03 kg/m^2^, *p* = 0.916, *d* = −0.017), and WC (−0.4 cm, *p* = 0.713, *d* = −0.058) for this group. Similarly to CE, the BC group had no significant changes over time (weight = 0.8 kg, *p* = 0.193, *d* = 0.221; BMI = 0.2 kg/m^2^, *p* = 0.221, *d* = 0.208; body fat = −0.4%, *p* = 0.479, *d* = −0.120; and WC = −0.1 cm, *p* = 0.950, *d* = −0.011). The groups’ comparison showed that CE was significantly different than BC in body mass index. No other significant difference among groups was observed.

#### 3.2.2. Cardiovascular Measures

**Figure 3:**
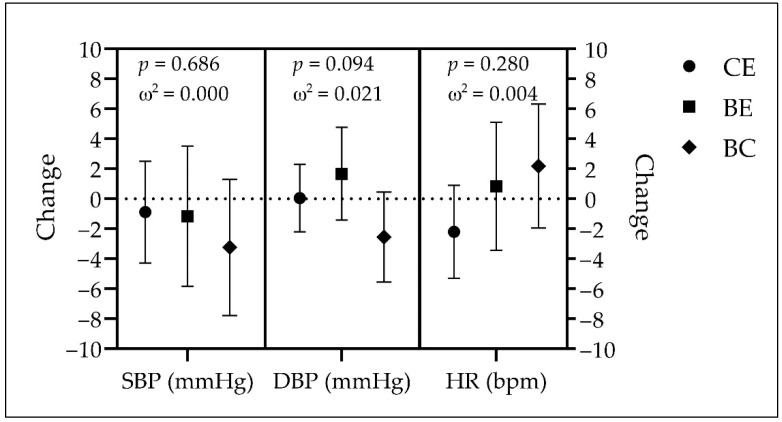
Changes over time in SBP, DBP, and HR. Data expressed as least squares mean and 95% confidence intervals; *p*-values calculated with one-way ANCOVA for group comparisons (adjusted for age, sex, marital status, income, and employment); omega squared (ω^2^) as effect size. BC: Brazilian control group (diamonds); BE: Brazilian experimental group (squares); CE: Canadian experimental group (circles); DBP: diastolic blood pressure; HR: heart rate; SBP: systolic blood pressure.

The cardiovascular variables of each group, at baseline and after the intervention, are presented in Table 6. The differences in these variables over time are presented in Figure 3.

The CE group was found to have significantly higher systolic blood pressure than the BC group at the end but not before the intervention. For diastolic blood pressure, CE was significantly higher than BE at baseline, and higher than BC at the end of the intervention. No significant differences were observed among groups for heart rate at any time point.

The groups showed no significant changes over time: CE (SBP = −0.9 mmHg, *p* = 0.607, *d* = −0.037; DBP = 0.1 mmHg, *p* = 0.959, *d* = 0.004; HR = −2.2 bpm, *p* = 0.163, *d* = −0.102), BE (SBP = −1.2 mmHg, *p* = 0.625, *d* = −0.076; DBP = 1.7 mmHg, *p* = 0.284, *d* = 0.168; HR = 0.8 bpm, *p* = 0.697, *d* = 0.061), and BC (SBP = −3.2 mmHg, *p* = 0.161, *d* = −0.238; DBP = −2.5 mmHg, *p* = 0.096, *d* = −0.283; HR = 2.2 bpm, *p* = 0.296, *d* = 0.177). There were no significant differences among groups.

#### 3.2.3. Physical Performance

**Figure 4:**
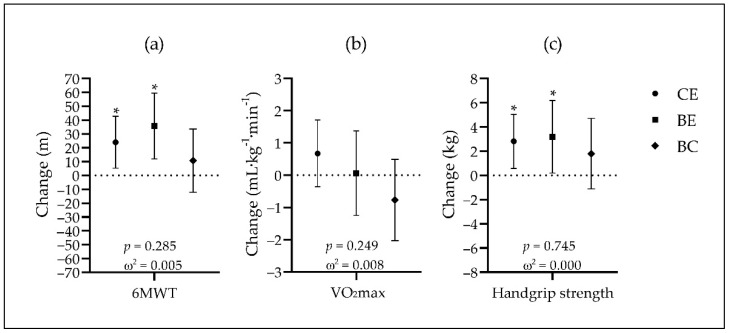
Changes over time in 6MWT (**a**), VO_2_max (**b**), and handgrip strength (**c**). Data expressed as least squares mean and 95% confidence intervals; *p*-values calculated with one-way ANCOVA for group comparisons (adjusted for age, sex, marital status, income, and employment); omega squared (ω^2^) as effect size. * Denotes a significant change over time (*p* < 0.05). 6MWT: six-minute walk test; BC: Brazilian control group (diamonds); BE: Brazilian experimental group (squares); CE: Canadian experimental group (circles); VO_2_max: maximal oxygen uptake.

The aerobic and musculoskeletal fitness variables of each group, at baseline and post-assessments, are shown in Table 7. The differences in these variables over time are presented in Figure 4.

No significant differences were observed among groups for any performance variable at either time point. 

Over time, participants in CE showed significant increases of 24.0 m in the 6MWT (*p* = 0.013, *d* = 0.216) and 2.8 kg in the handgrip strength (*p* = 0.014, *d* = 0.201), as well as a nonsignificant increase of 0.7 mL·kg^−1^·min^−1^ in VO_2_max (*p* = 0.930, *d* = 0.111). Participants in BE showed significant increases of 35.7 m in the 6MWT (*p* = 0.004, *d* = 0.464), and 3.2 kg in the handgrip strength (*p* = 0.039, *d* = 0.328), whereas VO_2_max did not show a significant change over time (0.06 mL·kg^−1^·min^−1^, *p* = 0.328, *d* = 0.014). No significant changes were observed in BC (6MWT = 10.7 m, *p* = 0.357, *d* = 0.156; VO_2_max = −0.8 mL·kg^−1^·min^−1^, *p* = 0.228, *d* = −0.205; and handgrip strength = 1.8 kg, *p* = 0.225, *d* = 0.206). There were no significant differences among groups.

## 4. Discussion

In line with a research need established by the World Health Organization, the Brazilian version of the ACCELERATION program was developed to make this intervention available to the Brazilian population, in a culturally appropriate format. The analyses demonstrate that the adapted intervention promoted significant improvements in some health and fitness outcomes, confirming our primary hypothesis. However, our secondary hypothesis was not confirmed, because although overall BE presented similar findings to CE, these results were not better than those of BC. The changes in BE were significant over time, but not large enough to be considered better than the control group.

Given the health disparities between populations across the globe, there have been multiple calls for culturally appropriate interventions, particularly in LMICs, which are grossly underrepresented in the literature [10,12,67]. This includes widespread calls to adapt interventions that have demonstrated efficacy and/or effectiveness in a culturally appropriate manner for LMICs [13,68,69]. Accordingly, recent studies have been conducted to perform a cross-cultural adaptation of interventions focusing on health behaviors. However, most studies to date do not include a control group, making it difficult to ascertain the effectiveness of the cultural adaptations [70,71,72]. Two studies [73,74] with some similarities to the present research did have a control group and also found improvements only in the experimental group. A chronic disease prevention intervention from Finland was culturally adapted to South Asians from India and Pakistan, in which participants had 15 sessions with a dietitian over a period of three years, addressing not only dietary aspects, but also physical activity promotion [73]. The other study was a 12-month internet-based program from the United States, tailored for Hispanic populations in the country, with a web diary and instructional videos focusing on physical activity and diet [74]. These studies adopted some of the same strategies used in the present research, such as educational sessions and self-regulation, as well as targeting more than one health behavior. Additionally, both interventions had considerably longer durations, which suggests that 12 weeks may also be an appropriate period to yield effective results. 

### 4.1. Demographics

All differences observed in demographic characteristics among groups were statistically significant. Although CE had more women than BE, female participants were the majority in all three groups, a proportion also found in other lifestyle management studies [75,76,77]. Regarding marital status, while again CE was different than BE, in the three groups, most of the participants were married. Given the higher household income expenditure of families in comparison to those of single individuals, married individuals may not consider adopting healthy behaviors a priority [78]. Therefore, the fact that the current program was offered for free possibly encouraged those who were married to participate in the intervention [79].

The CE group had more participants earning more than CAD 100,000/year than BE, and both Brazilian groups had more individuals earning less than CAD 50,000/year than CE, which is likely related to the fact that CE had more full-time employed individuals and fewer unemployed participants than BE and BC. Although it could be assumed that the higher rate of unemployment and the lower rate of full-time employment were responsible for the Brazilian individuals taking part in the program, the data about transport show the opposite. Both BE and BC had more participants than CE taking longer than 60 min to travel to the facility in charge of the program, and more participants in CE than BE and BC took less than 30 min for these trips. Further, more participants in CE than in BC used private transport, while more participants in the Brazilian groups travelled by transit, which shows the willingness and effort of the Brazilian individuals to participate in the intervention. In fact, many of these individuals did not live in the same city of the university where the assessments were carried out, and although there is an approved plan to build a rapid transit system to the university, currently, the only transit access to it is by bus, which can be a long commute [80]. 

### 4.2. Health and Fitness Measures

#### 4.2.1. Body Composition

Even though the intervention did not induce a change in body weight or BMI in BE, it did lead to a decrease of 3.3% (1.2%/36.0%) in body fat. Additionally, it is possible that this group also had an increase in fat-free mass, which would explain why body weight did not change in BE. However, as we did not assess fat-free mass in this study, it should be considered for future research to explore other possible benefits of this program on body composition. Similarly, we did not observe a change in WC, which may indicate that the program would not be effective in reducing abdominal/visceral fat. It is also possible that we did not see a difference in WC (a proxy of abdominal fat) because the change in body fat was relatively low (small to medium effect size), and changes in specific zones would be more difficult to observe.

The body composition results observed in BE are equivalent to those found in a meta-analysis with online interventions focused on health behavior change [81]. The same items were analyzed (weight, BMI, body fat percentage, and WC). No variable in the experimental groups presented better results than the control groups, while one study observed an improvement in body fat in the experimental group [82].

#### 4.2.2. Cardiovascular Measures

No changes in cardiovascular variables were observed for any group. It is possible that blood pressure, as well as heart rate, did not have significant decreases because the groups had normal values in all variables before the intervention [83].

A meta-analysis with studies using technology support such as websites to deliver physical activity and healthy eating programs [84] found contrasting results. Most of the included studies lasted ≥ 6 months, and overall, there was an improvement over time in SBP, DBP, and HR. A probable explanation for the difference between the findings from this review to those from the present research is that longer interventions promote better results in terms of physiological changes [85,86,87].

#### 4.2.3. Physical Performance

While no change was observed in VO_2_max, both experimental groups improved the distance covered in the 6MWT, with increases of 5.8% in BE and 3.9% in CE. Regarding handgrip strength, again both experimental groups improved their values, with increases of 5.1% in BE and 4.5% in CE. It is possible that the physical activity volume during the intervention was not enough to induce a significant change in VO_2_max. However, considering the improvement in walking distance and handgrip strength along with the decrease in body fat and a stable body weight, it seems possible that health-related physical fitness improved in concert with fat-free mass/skeletal muscle mass..

The results observed in BE are equivalent to or better than those found in studies with similar characteristics, focused on healthier lifestyles. The only study with such characteristics and which assessed the distance covered in the 6MWT, although without estimating VO_2_max, was a 12-week program, in which participants received an activity tracker and a tablet with an app to monitor their physical activity throughout the intervention, in addition to weekly counselling sessions over the phone [88]. No change in the 6MWT was observed. Regarding VO_2_max, again only one intervention was found, with similar characteristics to the present study. A two-month motivational program for runners, delivered via social media, in which the maximal consumption of oxygen was also estimated based on the results of a fitness test, reported that this variable did not change over time [89]. A systematic review on interventions focusing on chronic disease prevention through the use of technology support such as text messages and phone apps included one study that assessed handgrip strength in a 10-week program and did not observe changes after the intervention [90]. 

#### 4.2.4. Final Considerations

One of the innovations of the present study was the emphasis on some mechanisms of action of behavior change recently addressed in the literature, such as the perception of opportunity to translate intention into behavior [24,25,26]. Accordingly, the behavior change techniques used in the intervention focused not only on encouraging participants to make use of the best evidence to become healthier, but also on providing them with practical ways to do so, such as through the use of the elastic resistance band, which every participant received at the beginning of the program. Therefore, it is possible that the increase in muscular strength would not have been the same if we had not made use of this approach.

Alternatively, despite the positive results observed in the study, more social support could have contributed to better outcomes. Besides directions on how to build and increase a network of support, the intervention did provide some social support, namely through the weekly emails and the attention given during the assessments, mainly in the motivational interviewing session. However, unlike the original intervention, the adapted program was not delivered in person, and while Canadians tend to be more reserved, the wish to be close to other people is rooted in the Brazilian culture (and in Latino communities in general) [91]. In fact, some form of closer interaction between the participants and other individuals, for example, with the researcher and mainly with other participants, could have led to better results and would likely have been welcomed by those engaged in the program since sharing day-to-day experiences and feelings can critically affect wellbeing [92,93].

Indeed, according to Rhodes, Janssen, Bredin, Warburton, and Bauman [29], interventions based on more supervision, with intensive contact time, tend to be more effective. However, according to the same authors, such an approach demands funds and infrastructure that are frequently unavailable. This is particularly true for LMICs, where cost-effective alternatives that can benefit a large number of individuals are a pressing necessity [94,95].

### 4.3. Limitations

Unlike the original project, the adapted version of the program had a much smaller sample size, which limits generalization of the findings. While it would be impractical to obtain a similar number of participants given the very limited number of Brazilians living in the metropolitan area of Vancouver, the recruitment in partnership with the Brazilian consulate allowed the participation of residents from different cities in the region, contributing to an attenuation of the limitations related to the sample size. Additionally, this sample presented different demographic characteristics than the original program, which prevented a comparison with the entire Canadian cohort. Although different measures were taken to allow a proper adaptation of the program, it is possible that cultural differences as well as the different adherence rates may have led to some bias. Moreover, participants were not required to inform on their level of education, which prevented the examination of a variable that could have a significant bearing on the results. Additionally, some results were likely affected by the difference in the sample sizes. However, using the effect sizes to compare the samples allowed a fair examination of the similarities and differences among the groups. Another limitation was the design of the Brazilian trial, which was not truly randomized. Nonetheless, almost no differences were observed between the experimental and the control group at baseline, thus conferring a high level of reliability of the findings.

## 5. Future Directions 

The results of the present study may contribute to the promotion of healthier lifestyle behaviors in LMICs such as Brazil. Future studies should aim to have larger sample sizes and a complete randomized sampling for a more accurate replication of the findings. Notably, a follow-up study should be conducted with Brazilians living in Brazil, to control for contextual factors. Additionally, the examination of a follow-up period will show whether the changes observed in the intervention are maintained.

## 6. Conclusions

In line with a research priority recently established by the World Health Organization, a pan-Canadian chronic disease prevention intervention was adapted for Brazilians. The findings of the study demonstrate that the Brazilian version of the program yielded improvements in health-related physical fitness outcomes, such as body composition, walking distance, and muscular strength. According to these results, a scaled-up initiative based on this translated and culturally adapted intervention may contribute to the prevention of chronic diseases in Brazilians.

## Figures and Tables

**Table 1:** Topic, content, behavior change techniques, and respective mechanisms of action of educational videos.

Week/Video Title	Content	Behavior Change Technique (Mechanism of Action)
One: Physical activity—the basics	How to exercise in a safe and effective manner (FITT principle: frequency, intensity, time/duration, and type of activity)	-Information about health consequences (knowledge; beliefs about consequences) -Instruction on how to perform a behavior (knowledge; skills)
Two: Introduction to healthy eating	Principles established by the World Health Organization for a healthy diet	-Information about health consequences (knowledge; beliefs about consequences)
Three: Physical activity safety	Physical activity—what to avoid Exercising when sick Cold/hot weather	-Information about health consequences (knowledge; beliefs about consequences) -Instruction on how to perform a behavior (knowledge; skills)
Four: Golden rule of healthy eating	Food processing (prioritize unprocessed or minimally processed foods, limit processed foods, and avoid ultra-processed ones)	-Information about health consequences (knowledge; beliefs about consequences) -Restructuring the physical environment (behavioral cueing; environmental context/resources)
Five: Resistance training and aspects of general physical conditioning	Importance of resistance training and further explanation on how to do it, as well as the main concepts of fitness	-Instruction on how to perform a behavior (knowledge; skills) -Demonstration of the behavior (social learning/imitation)
Six: Fruits and vegetables	Importance of fruits and vegetables, and how to increase their consumption	-Instruction on how to perform a behavior (knowledge; skills)
Seven: Stress and coping	Chronic and persistent stress as a risk factor Coping—exercise, meditation, deep breathing	-Information about health consequences (knowledge; beliefs about consequences) -Instruction on how to perform a behavior (knowledge; skills) -Demonstration of the behavior (social learning/imitation)
Eight: Mindfulness eating	How to eat mindfully	-Instruction on how to perform a behavior (knowledge; skills)
Nine: Progression and barriers	How to progress and how to overcome barriers	-Graded tasks (beliefs about capabilities) -Problem solving/coping planning (beliefs about capabilities) -Prompts/cues (memory, attention, and decision processes) -Remove aversive stimulus (environmental context/resources)
Ten: Reading food labels	How to make healthy choices	-Instruction on how to perform a behavior (knowledge; skills)
Eleven: Dealing with setbacks	Relapsing is normal Coping strategies	-Relapse prevention (beliefs about capabilities) -Problem solving/coping planning (beliefs about capabilities)
Twelve: Wrapping up—graduation	Long term positive health behaviors	-Habit formation (behavioral cueing) -Feedback on behavior (motivation) -Goal setting (behavior)

**Table 2:** Main topic and overall content, as well as behavior change techniques and respective mechanisms of action used in each individualized email.

Week/Email Main Topic	Content	Behavior Change Technique (Mechanism of Action)
One: Motivation	-Fostering autonomy, with empathy and reminders of realistic goals -Listing positive expected outcomes and inquiring about benefits from the specific plan	-Information about health consequences (knowledge; beliefs about consequences) -Focus on past success (beliefs about capabilities) -Review behavior goals (goals)
Two: Small changes—big outcomes	-Strategies to progress safely and successfully -Benefits of small increments of physical activity, small improvements in fruits/vegetables consumption, and small decreases in smoking	-Graded tasks (beliefs about capabilities) -Restructuring the social environment (environmental context/resources) -Restructuring the physical environment (behavioral cueing; environmental context/resources)
Three: Habit formation	-Cues and prompts to elicit behavior change -Consistency of practices -Eating well on a budget -Provision of positive feedback and encouragement	-Prompts/cues (memory, attention, and decision processes) -Remove aversive stimulus (environmental context/resources) -Habit formation (behavioral cueing) -Feedback on behavior (motivation)
Four: Avoiding relapse	-Identifying and planning to overcome potential challenges to translate intentions into actions -Revising goals and plans/assistance on problem solving	-Relapse prevention (beliefs about capabilities) -Review behavior goals (goals) -Problem solving/coping planning (beliefs about capabilities)
Five: Enjoyable physical activity	-Guidance on accessing and engaging in fun physical activity -Traditional and less conventional formats and environments	-Instruction on how to perform a behavior (knowledge; skills) -Information about emotional consequences (beliefs about consequences) -Restructuring the social environment (environmental context/resources) -Restructuring the physical environment (behavioral cueing; environmental context/resources)
Six: Halfway assessment	-Guidance on how to do a simple and effective health and fitness assessment -Provision of positive feedback and encouragement	-Review behavior goals (goals) -Feedback on behavior (motivation)
Seven: Easy healthy and happy eating	-Tasty, practical, and inexpensive meal and recipe suggestions -Seasonal food availability	-Instruction on how to perform a behavior (knowledge; skills) -Information about emotional consequences (beliefs about consequences)
Eight: Self-regulation	-Self-monitoring—reinforcement on record keeping of daily steps/MVPA, fruit/vegetable intake, reduction in smoking -Revising goals and plans/assistance on problem solving	-Self-monitoring of behavior (behavioral regulation) -Review behavior goals (goals) -Problem solving/coping planning (beliefs about capabilities)
Nine: Time management	-Adjusting priorities -Overcoming barriers to eat more produce -Provision of positive feedback and encouragement	-Action planning (behavioral cueing; behavioral regulation) -Feedback on behavior (motivation)
Ten: Social support beyond the program	-Guidance on proactivity to identifying and establishing a social net of support, and increasing confidence	-Social support (social influences; environmental context/resources) -Restructuring the social environment (environmental context/resources)
Eleven: Sit less and move more	-Why sitting can be detrimental to health -How to turn sedentary activities into light physical activities	-Information about health consequences (knowledge; beliefs about consequences) -Salience of consequences (beliefs about consequences) -Restructuring the physical environment (behavioral cueing; environmental context/resources)
Twelve: Onward and upward	-Reviewing key aspects of the program	-Review behavior goals (goals) -Feedback on behavior (motivation) -Problem solving/coping planning (beliefs about capabilities)

**Table 3:** Adaptation of resources for data collection and delivery of the intervention.

Resource	Cultural Adaptation
Data collection	
Pre-participation screening	Since the original PAR-Q+ was developed with the Canadian population, a culturally adapted version of the questionnaire was validated for the Brazilian population.
Health behavior questionnaires	Some items in the questionnaires used in the Canadian program were not common in the context of the Brazilian culture. Therefore, they were replaced by equivalent elements in the Brazilian tradition.
Intervention	
Educational videos	Preliminary consultation with members of the targeted population indicated the desire for more educational sessions about healthy diet as well as the vast use of context-rich images to help to convey the messages of the program. To address this wish, almost half of the videos addressed food-related content.
Individualized emails	Several participants were newcomers to Canada. Consequently, most of the Brazilian participants had a clear desire to have social support through this online interaction, instead of only receiving guidance on different aspects of the program. Such a reality required a more flexible approach from the research team, to promote reception while diligently delivering all components of the intervention. This included providing direction on how to find basic sets of Brazilian foods as well as on how to engage in more familiar types of physical activity, such as soccer and recreational martial arts.

**Table 4:** Demographic data compared among groups. Data expressed as frequency counts (percentage), except for age, expressed as mean ± standard deviation.

	*n* ^1^	CE	BE	BC	*p*-Value	Effect Size
Age (years)	194/41/35	47.5 ± 9.3 ^†‡^	34.9 ± 6.4	36.2 ± 6.9	<0.001 ^2^	0.279 ^4^
Sex	194/41/35				0.001 ^3^	0.230 ^5^
Female		168 (86.6) ^†^	26 (63.4)	25 (71.4)		
Male		26 (13.4) ^†^	15 (36.6)	10 (28.6)		
Marital status	189/41/35				0.012 ^3^	0.182 ^5^
Married		140 (74.1) ^†^	39 (95.1)	28 (80.0)		
Not married		49 (25.9) ^†^	2 (4.9)	7 (20.0)		
Income	176/40/34				0.001 ^3^	0.209 ^6^
<CAD 50,000/year		32 (18.2) ^†‡^	15 (37.5)	16 (47.1)		
CAD 50,000 to 74,999/year		36 (20.4)	10 (25.0)	7 (20.6)		
CAD 75,000 to 99,999/year		39 (22.2)	7 (17.5)	7 (20.6)		
>CAD 100,000/year		69 (39.2) ^‡^	8 (20.0)	4 (11.8)		
Employment	128/41/35				<0.001 ^3^	0.253 ^6^
Full-time		103 (80.5) ^†‡^	23 (56.1)	14 (40.0)		
Part-time		10 (7.8) ^‡^	5 (12.2)	9 (25.7)		
Unemployed		15 (11.7) ^†‡^	13 (31.7)	12 (34.3)		
Transport time	128/41/35				<0.001 ^3^	0.459 ^6^
<30 min		86 (58.1) ^†‡^	3 (7.3)	4 (11.4)		
30 to 60 min		55 (37.2)	12 (29.3)	12 (34.3)		
>60 min		7 (4.7) ^†‡^	26 (63.4)	19 (54.3)		
Transport mode	131/39/34				<0.001 ^3^	0.422 ^5^
Private transport		114 (87.0) ^‡^	22 (56.4)	14 (41.2)		
Public transit		17 (13.0) ^†‡^	17 (43.6)	20 (58.8)		

^1^ Sample sizes for CE/BE/BC, respectively, for each variable; ^2^ *p*-value calculated with one-way ANOVA with Welch’s correction; ^3^ *p*-value calculated with X^2^ test of independence; ^4^ effect size calculated as omega squared (ω^2^); ^5^ effect size calculated as phi (φ); ^6^ effect size calculated as Cramer’s V. ^†^ Denotes a significant difference from BE within categories (*p* < 0.05); ^‡^ denotes a significant difference from BC within categories (*p* < 0.05). BC: Brazilian control group; BE: Brazilian experimental group; CE: Canadian experimental group.

**Table 5:** Body composition variables compared among groups over time. Data expressed as least squares mean ± SEM.

Variable	*n* ^1^	Time Point	CE	BE	BC	*p*-Value ^2^
Weight (kg)	188/41/35	Pre	79.8 ± 2.8	81.5 ± 3.8	79.1 ± 3.7	0.871
		Post	79.6 ± 2.7	81.6 ± 3.7	79.9 ± 3.5	0.902
BMI (kg/m^2^)	188/41/35	Pre	28.7 ± 1.0	29.7 ± 1.4	27.5 ± 1.3	0.444
		Post	28.6 ± 1.0	29.6 ± 1.3	27.7 ± 1.3	0.524
Body fat (%)	188/41/35	Pre	33.4 ± 1.2	36.0 ± 1.7	31.7 ± 1.6	0.124
		Post	33.0 ± 1.2	34.8 ± 1.6	31.3 ± 1.5	0.217
WC (cm)	191/41/35	Pre	92.8 ± 2.3	98.1 ± 3.1	96.4 ± 3.0	0.435
		Post	93.8 ± 2.2	97.7 ± 3.0	96.4 ± 3.0	0.628

^1^ Sample sizes for CE/BE/BC, respectively, for each variable; ^2^ *p*-value calculated with one-way ANCOVA for comparisons among groups within the same time point (adjusted for age, sex, marital status, income, and employment). BC: Brazilian control group; BE: Brazilian experimental group; BMI: body mass index; CE: Canadian experimental group; WC: waist circumference.

**Table 6:** Cardiovascular variables compared among groups over time. Data expressed as least squares mean ± SEM.

Variable	*n* ^1^	Time Point	CE	BE	BC	*p*-Value ^2^
SBP (mmHg)	192/41/35	Pre	118.6 ± 2.4	111.1 ± 3.2	111.8 ± 3.2	0.168
		Post	117.7 ± 2.1 ^‡^	110.0 ± 2.8	108.6 ± 2.7	0.035
DBP (mmHg)	192/41/35	Pre	76.8 ± 1.5 ^†^	69.9 ± 2.0	72.5 ± 2.0	0.038
		Post	76.8 ± 1.3 ^‡^	71.6 ± 1.8	69.9 ± 1.7	0.009
HR (bpm)	191/41/35	Pre	72.3 ± 1.5	70.3 ± 2.0	71.6 ± 2.0	0.758
		Post	70.1 ± 1.6	71.2 ± 2.2	73.8 ± 2.2	0.417

^1^ Sample sizes for CE/BE/BC, respectively, for each variable; ^2^ *p*-value calculated with ANCOVA for comparisons among groups within the same time point (adjusted for age, sex, marital status, income, and employment). ^†^ Denotes a significant difference from BE within a time point (*p* < 0.05); ^‡^ denotes a significant difference from BC within a time point (*p* < 0.05). BC: Brazilian control group; BE: Brazilian experimental group; CE: Canadian experimental group; DBP: diastolic blood pressure; HR: heart rate; SBP: systolic blood pressure.

**Table 7:** Physical performance variables compared among groups over time. Data expressed as least squares mean ± SEM.

Variable	*n* ^1^	Time Point	CE	BE	BC	*p*-Value ^2^
6MWT (m)	139/41/35	Pre	611.3 ± 11.5	620.2 ± 14.6	618.1 ± 14.1	0.900
		Post	635.3 ± 9.8	655.9 ± 12.5	628.8 ± 12.0	0.206
VO_2_max (mL·kg^−1^·min^−1^)	135/41/35	Pre	33.6 ± 1.2	36.4 ± 1.5	36.4 ± 1.4	0.284
		Post	34.3 ± 1.1	36.5 ± 1.3	35.7 ± 1.3	0.499
Handgrip strength (kg)	154/41/35	Pre	61.6 ± 2.0	62.3 ± 2.7	67.0 ± 2.6	0.224
		Post	64.4 ± 1.8	65.5 ± 2.4	68.8 ± 2.3	0.319

^1^ Sample sizes for CE/BE/BC, respectively, for each variable; ^2^ *p*-value calculated with one-way ANCOVA for comparisons among groups within the same time point (adjusted for age, sex, marital status, income, and employment). BC: Brazilian control group; BE: Brazilian experimental group; CE: Canadian experimental group; 6MWT: six-minute walk test; VO_2_max: maximal oxygen uptake.

## Data Availability

Data are available upon reasonable request.
